# Supplementary material for: Polypyrrole-Coated Low-Crystallinity Iron Oxide Grown on Carbon Cloth Enabling Enhanced Electrochemical Supercapacitor Performance
Source: Molecules. 2023 Jan 3;28(1):434. doi: 10.3390/molecules28010434 (PMC9823998; doi:10.3390/molecules28010434)
Supplement: Supplementary file 1 [file molecules-28-00434-s001.zip › molecules-2116421-supplementary.pdf]

Supporting Information

Polypyrrole-coated low-crystallinity iron oxide grown on carbon cloth enabling enhanced electrochemical supercapacitor performance

Chunhui Wu, Zifan Pei, Menglin Lv, Duchen Huang, Yuan Wang,* Shaojun Yuan*

Low-carbon Technology & Chemical Reaction Engineering Lab, College of Chemical Engineering, Sichuan University, Chengdu 610065, China.

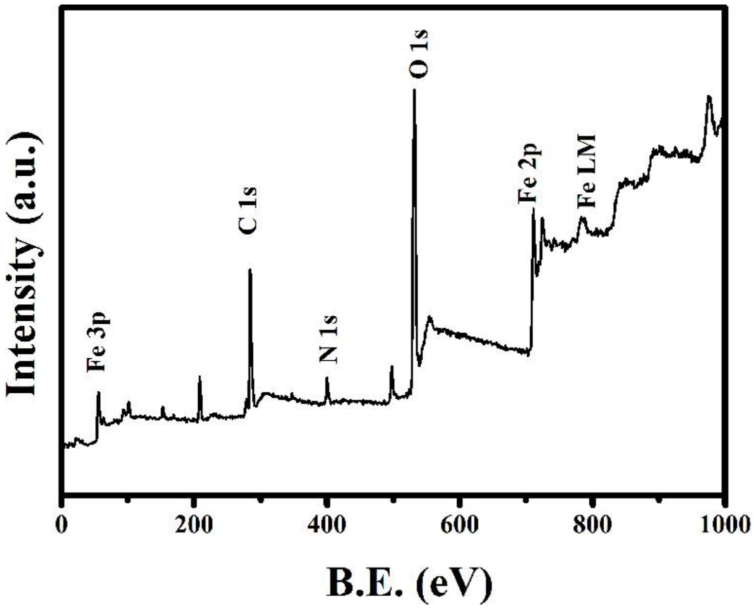

Figure S1. Wide scan XPS spectrum of D-Fe_2O_3@PPy/CC electrode.

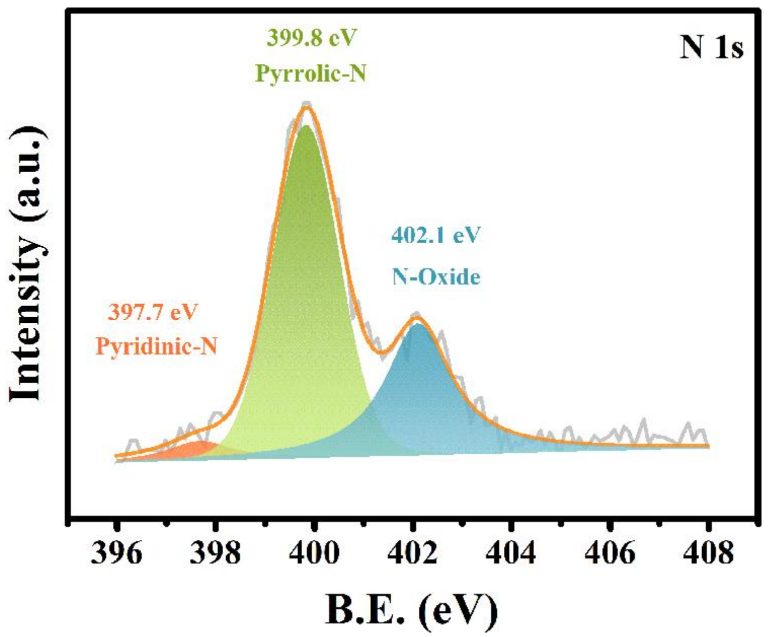

Figure S2. N 1s XPS spectrum of D-Fe_2O_3@PPy/CC electrode.

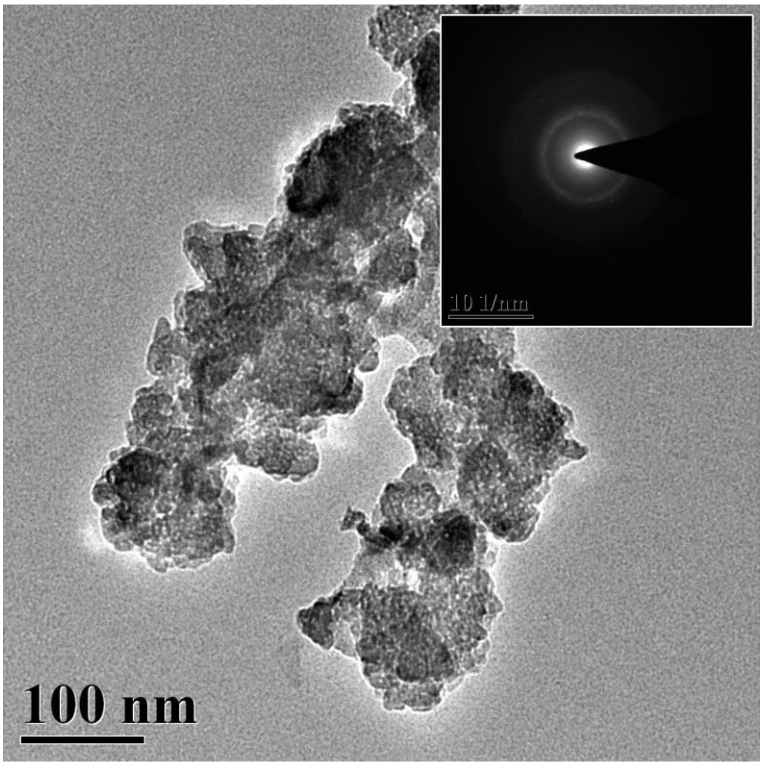

Figure S3. The TEM image of D-Fe_2O_3@PPy and the related SAED pattern.

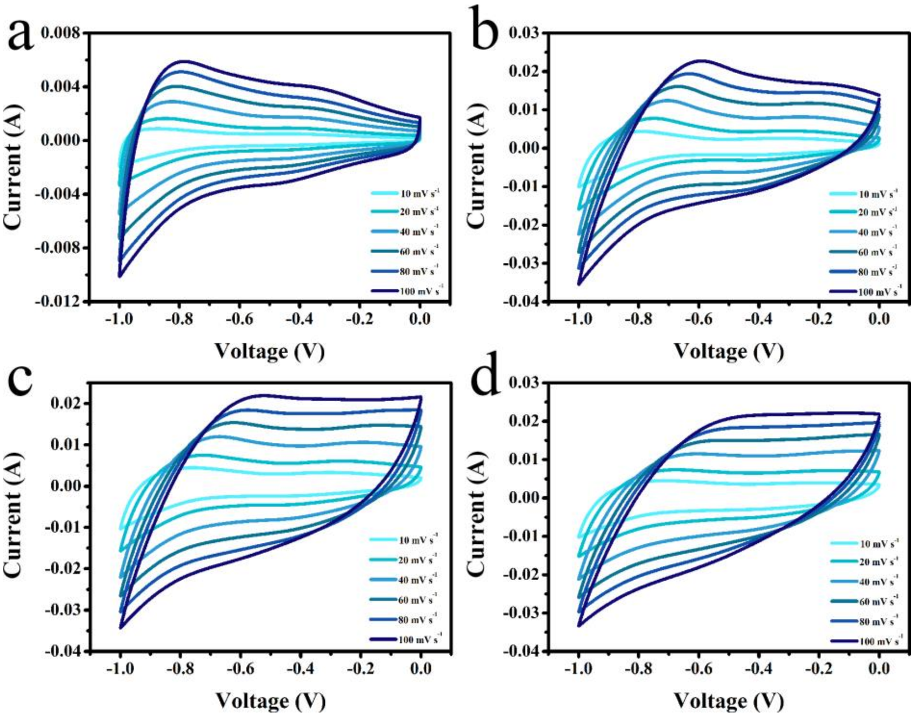

Figure S4. CV curves of D-Fe_2O_3/CC electrodes. (a) pristine Fe_2O_3, (b) D- Fe_2O_3-0.5h, (c) D- Fe_2O_3-1h, and (d) D- Fe_2O_3-4h.

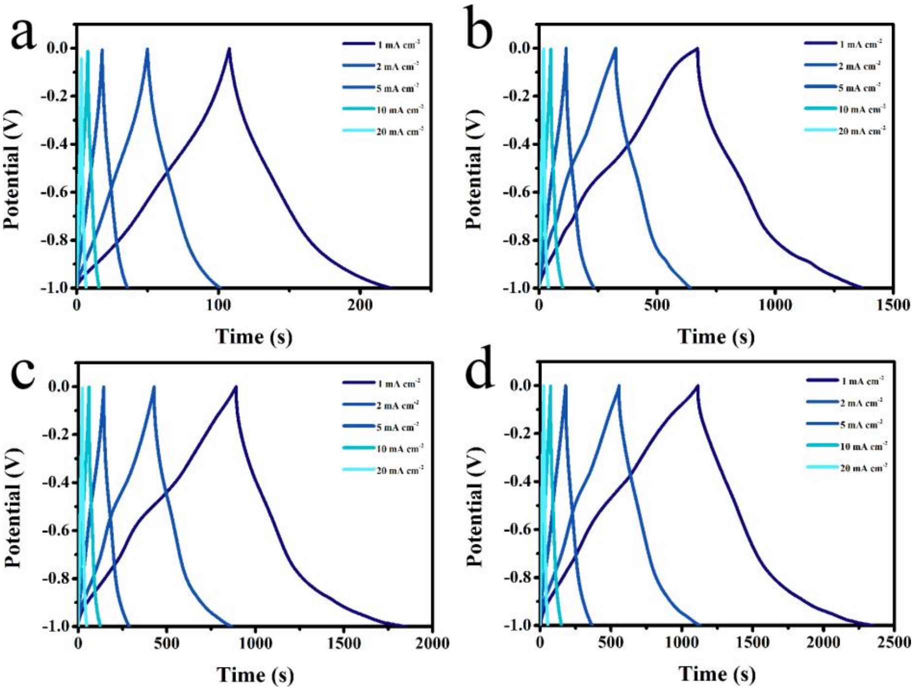

Figure S5. GCD curves of D-Fe_2O_3/CC electrodes. (a) pristine Fe_2O_3, (b) D- Fe_2O_3-0.5h, (c) D- Fe_2O_3-1h, and (d) D- Fe_2O_3-4h.

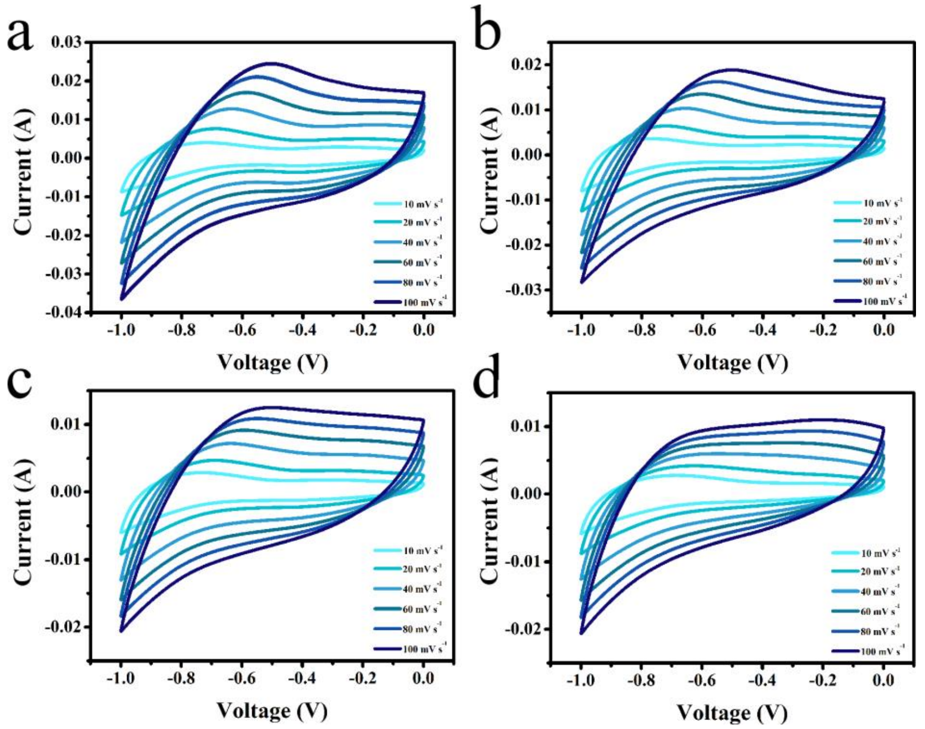

Figure S6. CV curves of D-Fe_2O_3@PPy/CC electrodes. (a) D-Fe_2O_3@PPy-30s, (b) D-Fe_2O_3@PPy-60s, (c) D-Fe_2O_3@PPy-120s, and (d) D-Fe_2O_3@PPy-180s.

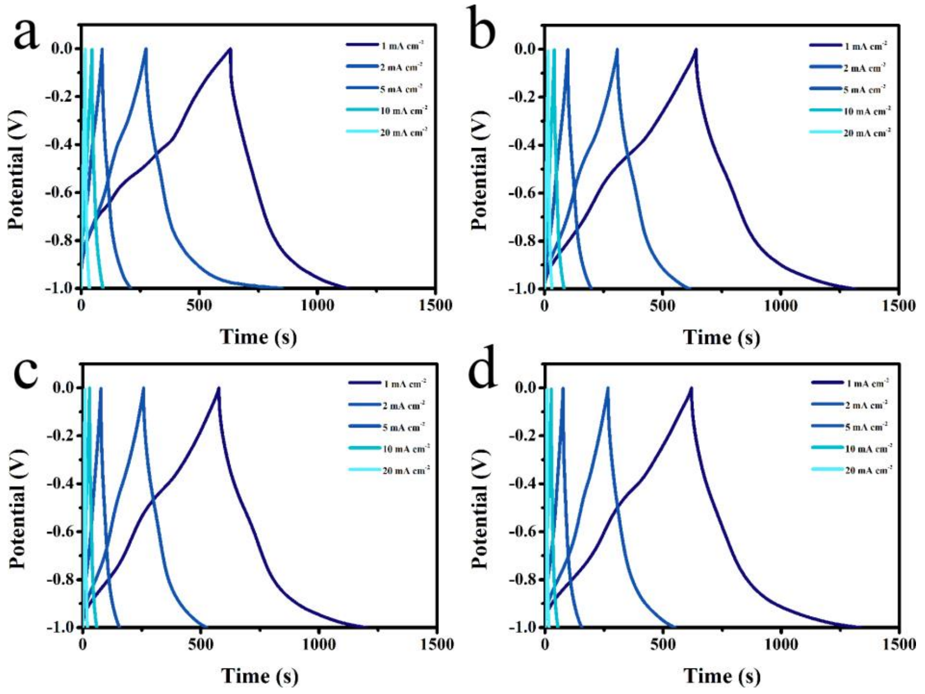

Figure S7. GCD curves of D-Fe_2O_3@PPy/CC electrodes. (a) D-Fe_2O_3@PPy-30s, (b) D-Fe_2O_3@PPy-60s, (c) D-Fe_2O_3@PPy-120s, and (d) D-Fe_2O_3@PPy-180s.

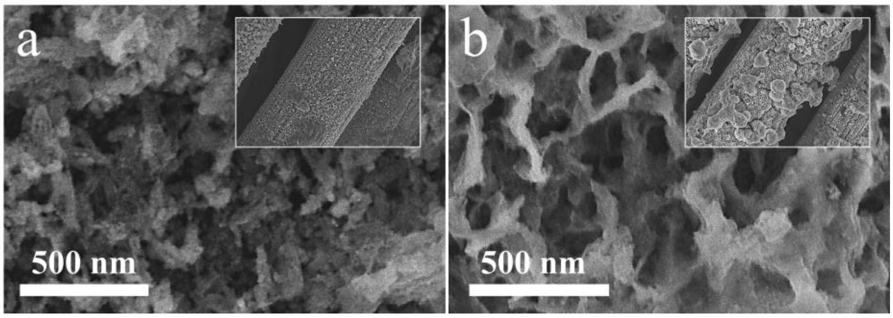

Figure S8. SEM images of (a) Fe_2O_3/CC and (b) D-Fe_2O_3@PPy/CC electrode after long cycling test.

Table S1. Electrochemical performance of Fe_2O_3-based and D-Fe_2O_3@PPy/CC anodes for supercapacitors.

Electrodes	Electrolyte	Potential (V)	Specific capacitance Rate capability	Ref.
**D-Fe_2O_3@PPy/CC**	**1 M Na_2SO_4**	**-1-0**	**615 mF cm^{-2} at 1 mA cm^{-2}**<br>**208 mF cm^{-2} at 20 mA cm^{-2}**	**This work**
**FeO-CNT-20/CC**	1 M Na_2SO_4	-1-0	483.4 mF cm^{-2} at 1 mA cm^{-2}	*ACS Appl. Mater. Interfaces* **2021**, 13, 45670.
**Fe_2O_3 NTRs@PPy/CC**	1 M Na_2SO_4	-1–0	237 mF cm^{-2} at 1 mA cm^{-2}<br>102 mF cm^{-2} at 20 mA cm^{-2}	*Electrochimica Acta* **2021**, *386*, 138486
**CC/Fe_2O_3@b-C/CC**	6 M KOH	-1.2–0	246.2 mC cm^{-2} at 5 mV s^{-1}	*J. Power Sources* **2021**,*489*,229501
**Fe_2O_3@N-C/CC**	4 M KOH	-1.0–0	183.3 mF cm^{-2} at 3 mA cm^{-2}	*ACS Appl. Energy Mater.* **2020**, *3,* 12162−12171
**$Fe_2O_{3-\delta}$ nanorod arrays/CC**	1 M LiOH	-0.9–0	350 mF cm^{-2} at 1 mA cm^{-2}	*Nano Energy* **2018**, *45,* 390–397
**Fe_2O_3 nanoflakes**	1M KOH	-1.2–-0.2	169.2 mF cm^{-2} at 1 mA cm^{-2}<br>61 mF cm^{-2} at 5 mA cm^{-2}	*Ceram. Int.* **2018**, *44*, 10635–10645
**Fe_2O_3-P nanorods/CC**	1M Na_2SO_4	-0.8–0	340 mF cm^{-2} at 1 mA cm^{-2}<br>112 mF cm^{-2} at 20 mA cm^{-2}	*Nano Energy* **2018**, *49*, 155–162
**α-Fe_2O_3 nanoarrays/CC**	1M Na_2SO_4	-0.8–0	207 mF cm^{-2} at 1 mA cm^{-2}<br>120.8 mF cm^{-2} at 6 mA cm^{-2}	*Angew. Chem. Int. Ed.* **2017**, *56*, 1105 –1110
**C@Fe_3O_4 nanosheets**	1M LiCl	-1–0	127 mF cm^{-2} at 1 mA cm^{-2}<br>60.74 mF cm^{-2} at 8 mA cm^{-2}	*J. Mater. Chem. A* **2016** *4*, 14877–14883
**α-Fe_2O_3 nanowires**	1 M Na_2SO_4	-0.8–0	103 mF cm^{-2} at 0.5 mA cm^{-2}<br>26.5 mF cm^{-2} at 5.7 mA cm^{-2}	*ACS Appl. Mater. Interfaces* **2015**, *7*, 14843−14850
**Fe_2O_3 nanotubes**	5 M LiCl	-0.8–0	180.4 mF cm^{-2} at 1 mA cm^{-2}<br>119.9 mF cm^{-2} at 10 mA cm^{-2}	*Nano Lett.* **2014**, *14*, 731–736
